# Supplementary material for: Copepods as Indicators of Different Water Masses during the Northeast Monsoon Prevailing Period in the Northeast Taiwan
Source: Biology (Basel). 2022 Sep 16;11(9):1357. doi: 10.3390/biology11091357 (PMC9495807; doi:10.3390/biology11091357)
Supplement: Supplementary file 1 [file biology-11-01357-s001.zip › biology-1877486-supplementary.pdf]

Supplementary: Figures S1–S4 the distribution patterns of surface water environmental parameter in the research area during our research.

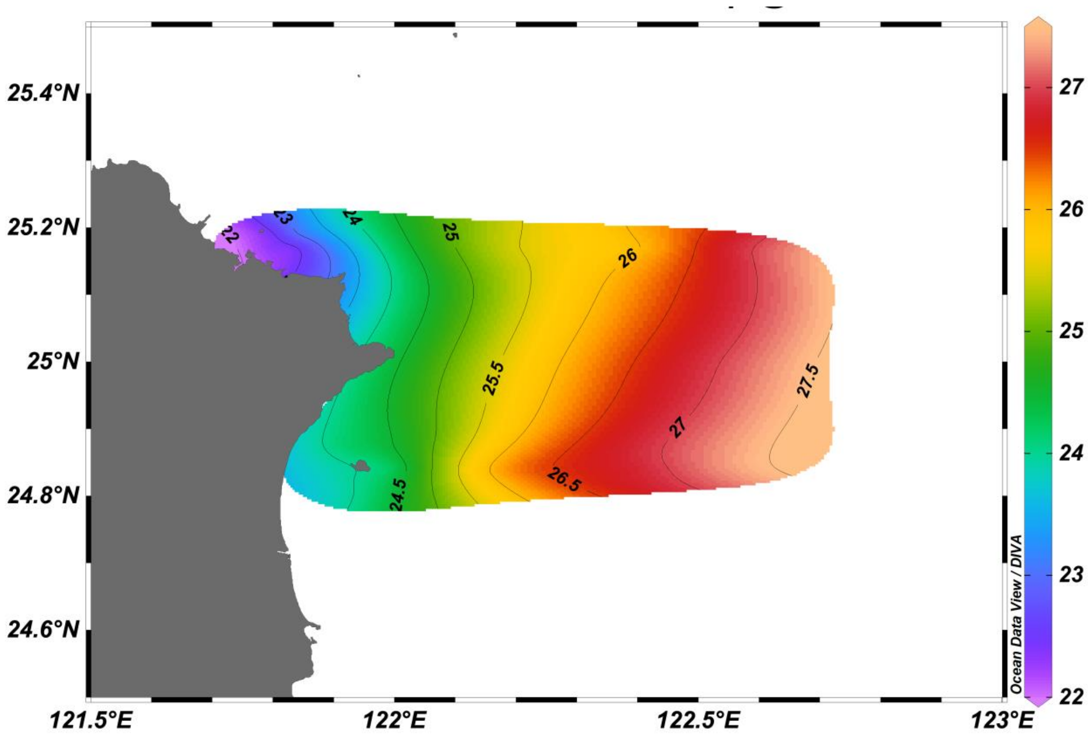

Figure S1. Horizontal distribution of surface water temperature in the research area during our research.

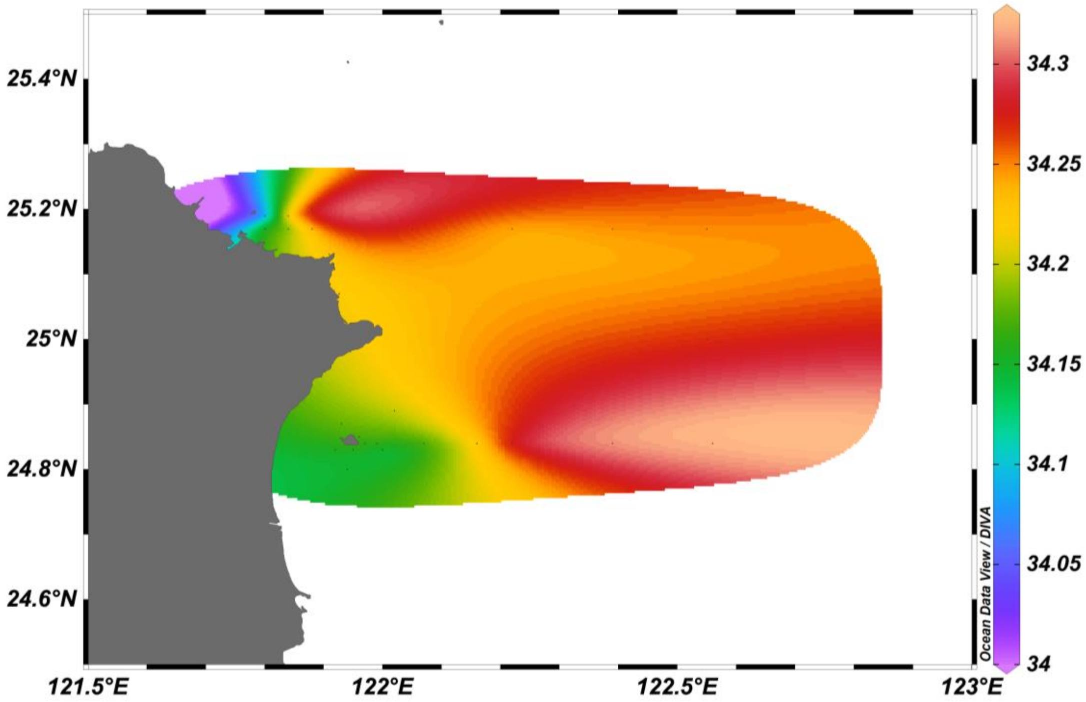

Figure S2. Horizontal distribution of surface water salinity in the research area during our research.

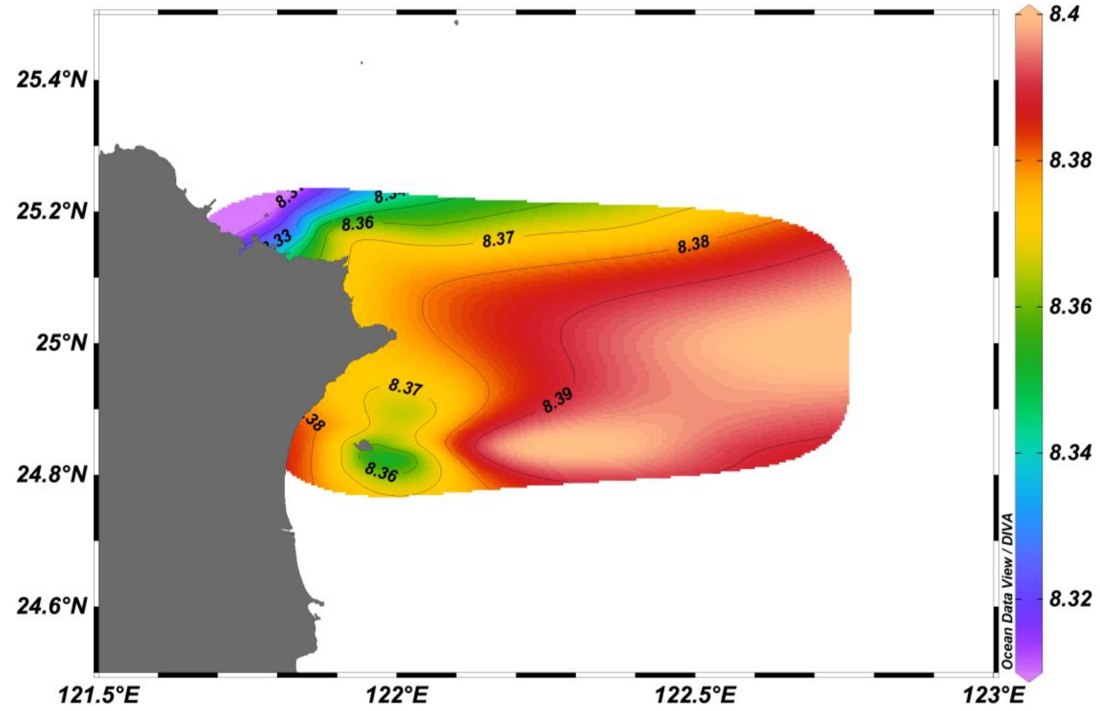

Figure S3. Horizontal distribution of surface water pH in the research area during our research.

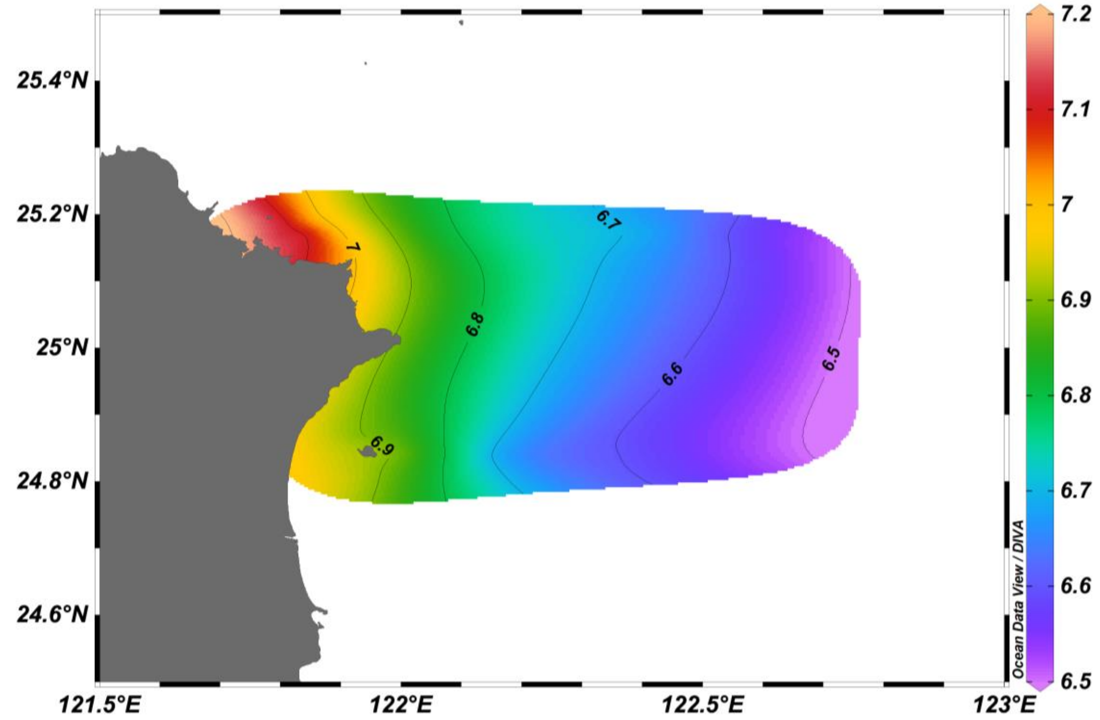

Figure S4. Horizontal distribution of surface water dissolved oxygen in the research area during our research.

Supplementary: Figures S5–S26 showing distribution patterns of indicator species in the research area during our research.

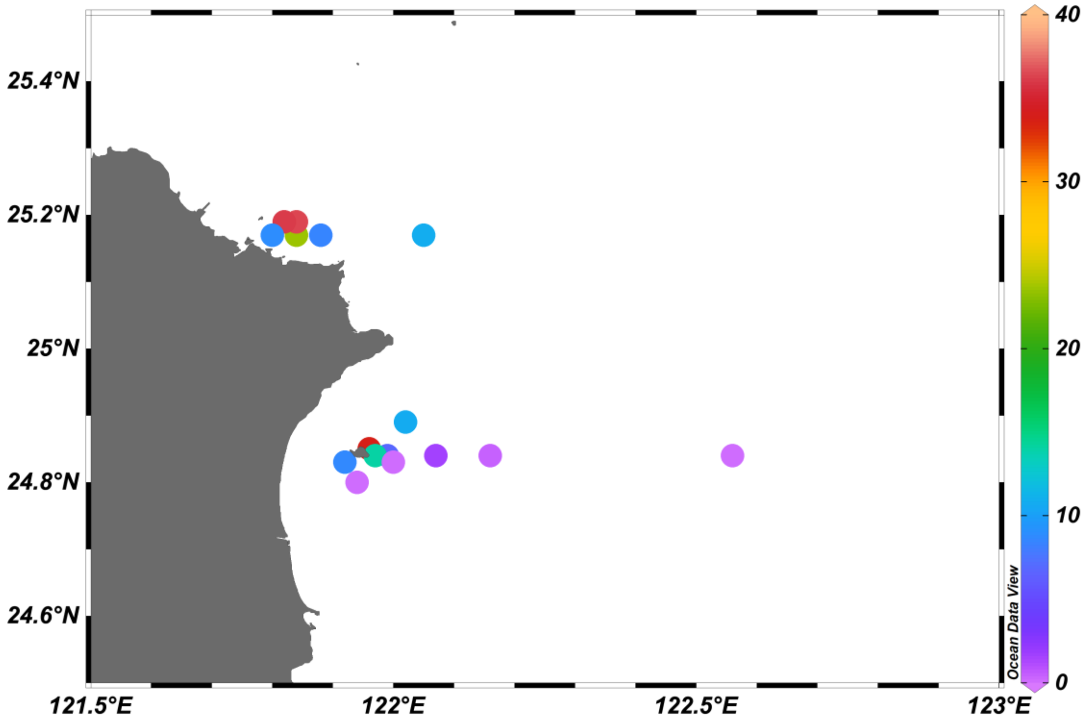

Figure S5. Distribution of *Acartia pacifica* in the research area during our research.

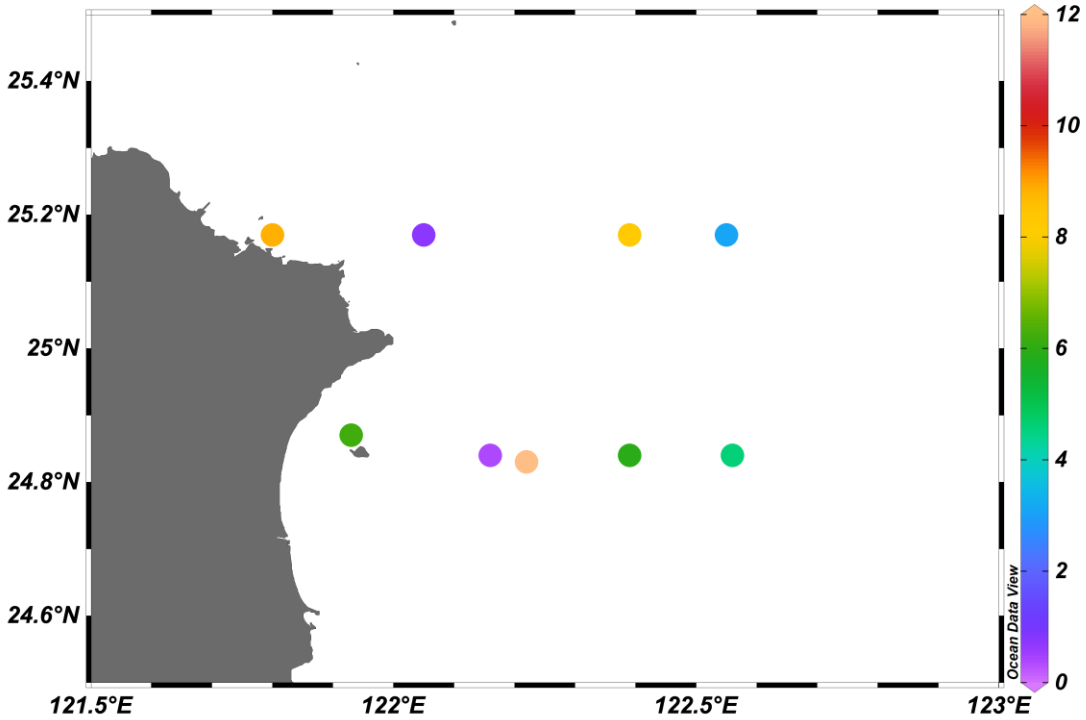

Figure S6. Distribution of *Acrocalanus monachus* in the research area during our research.

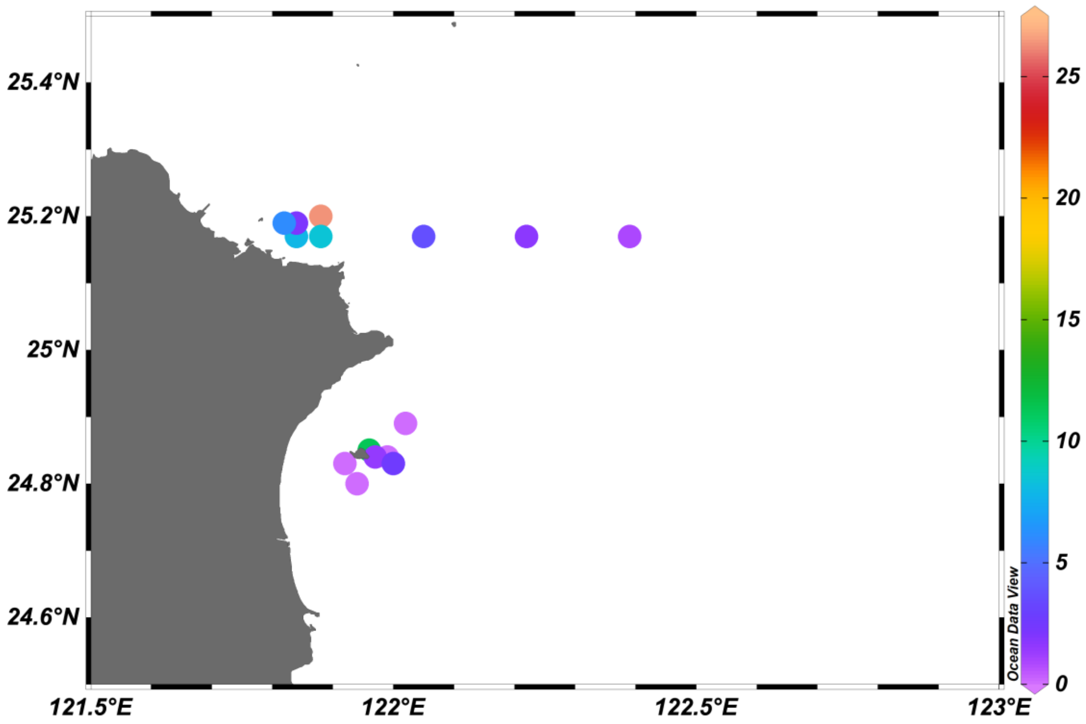

Figure S7. Distribution of *Calanopia elliptica* in the research area during our research.

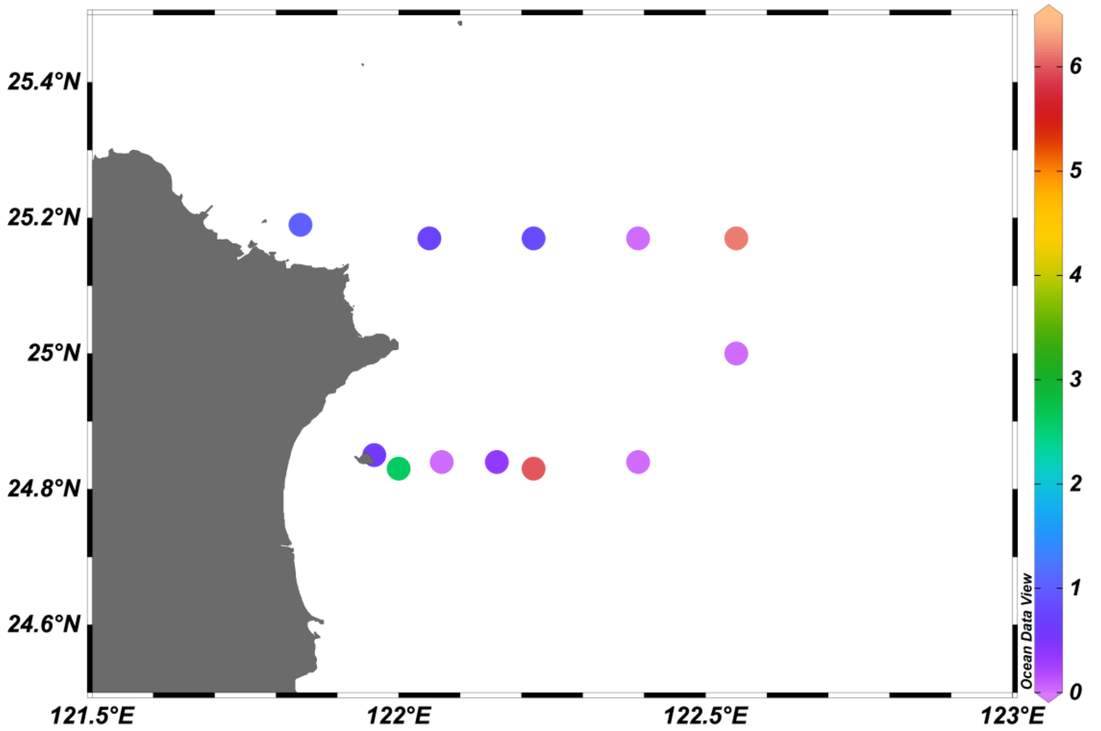

Figure S8. Distribution of *Candacia aethiopica* in the research area during our research.

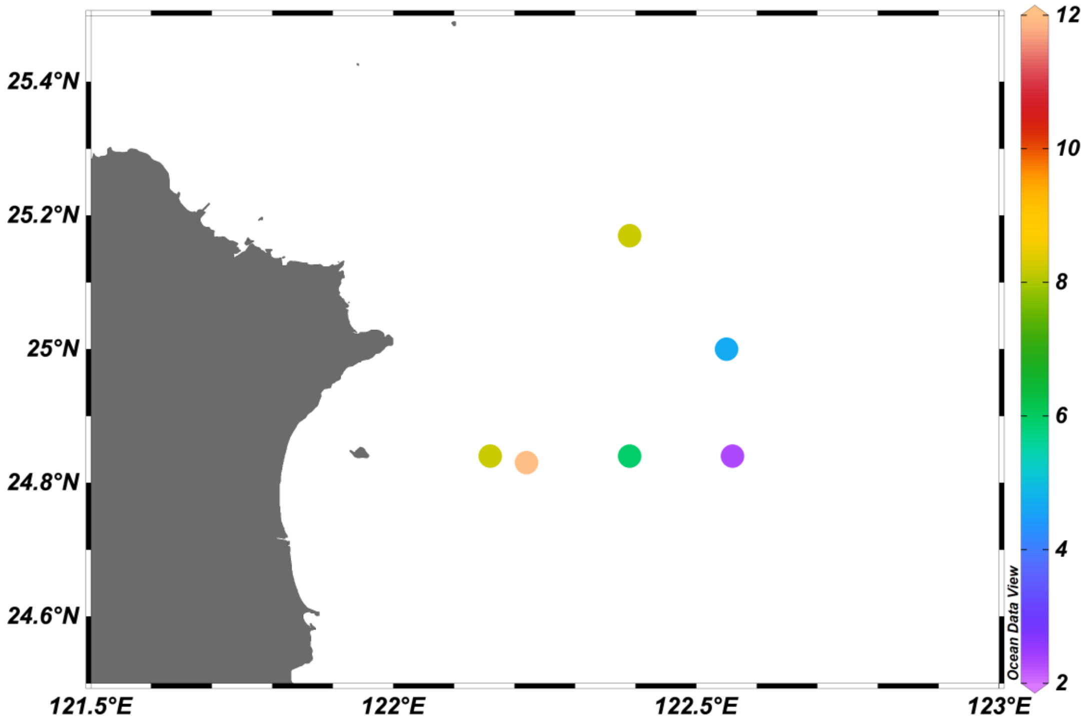

Figure S9. Distribution of *Candacia* copepodites in research area during our research.

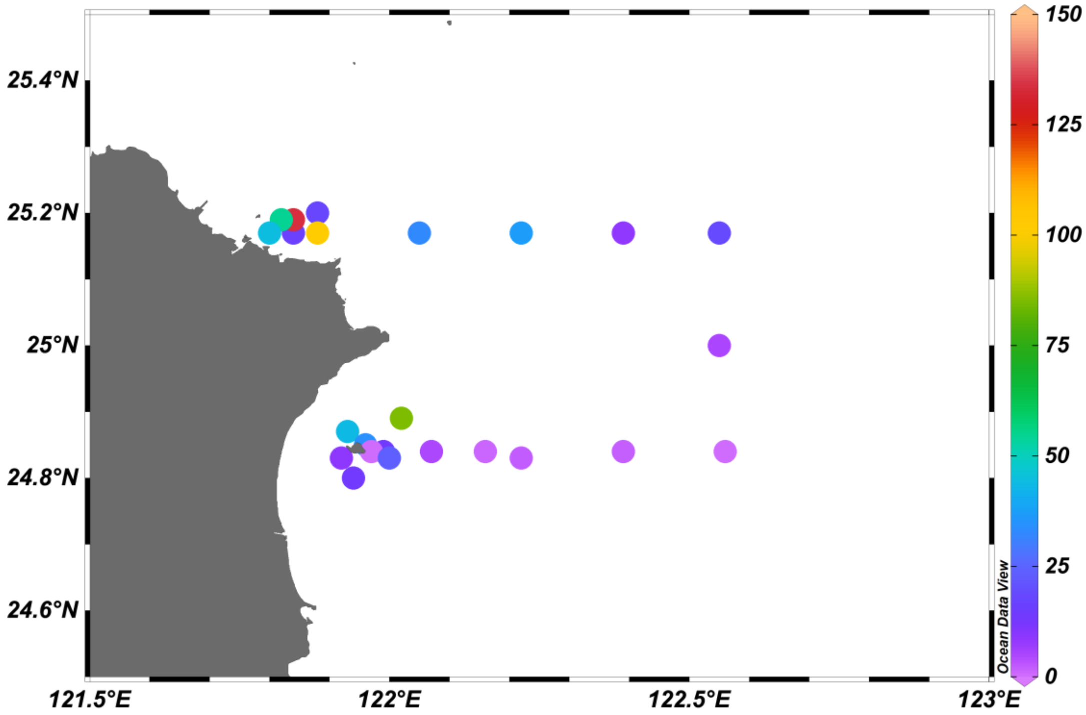

Figure S10. Distribution of *Canthocalanus pauper* in the research area during our research.

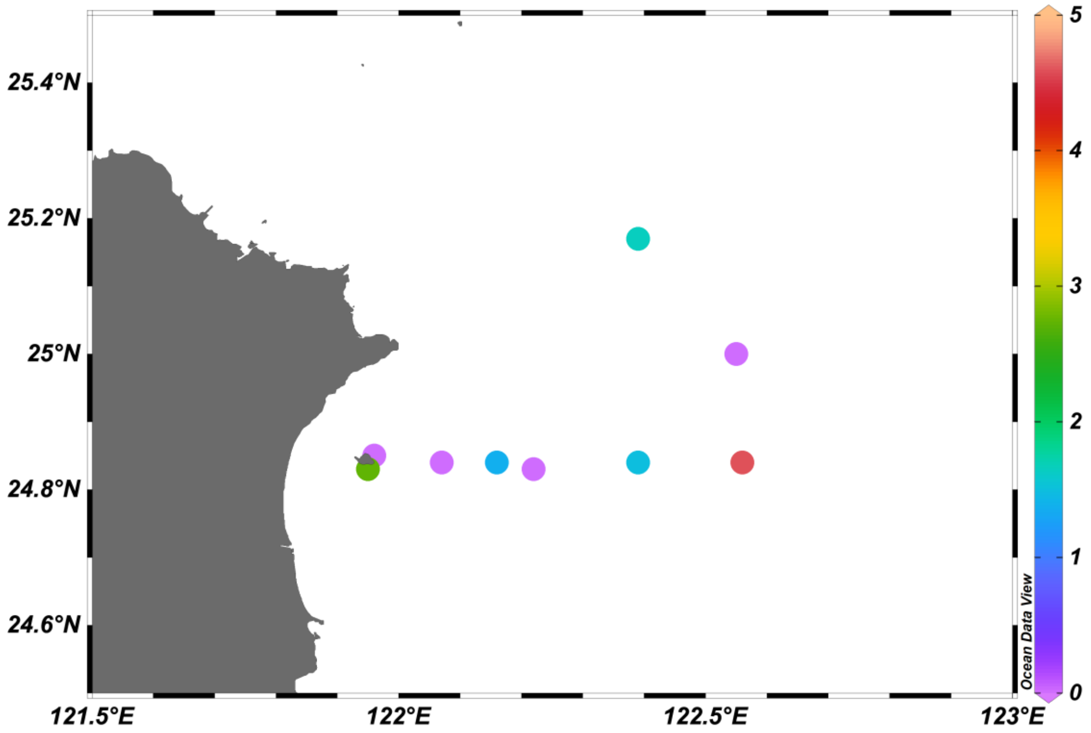

Figure S11. Distribution of *Centropages calaninus* in the research area during our research.

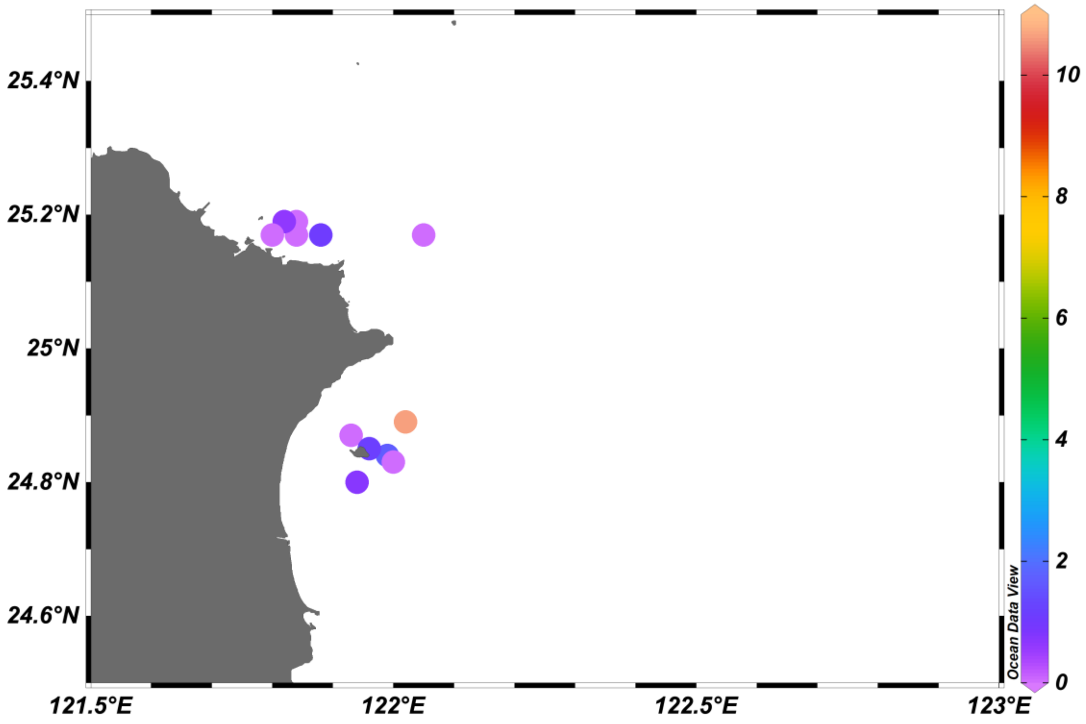

Figure S12. Distribution of *Centropages furcatus* in the research area during our research.

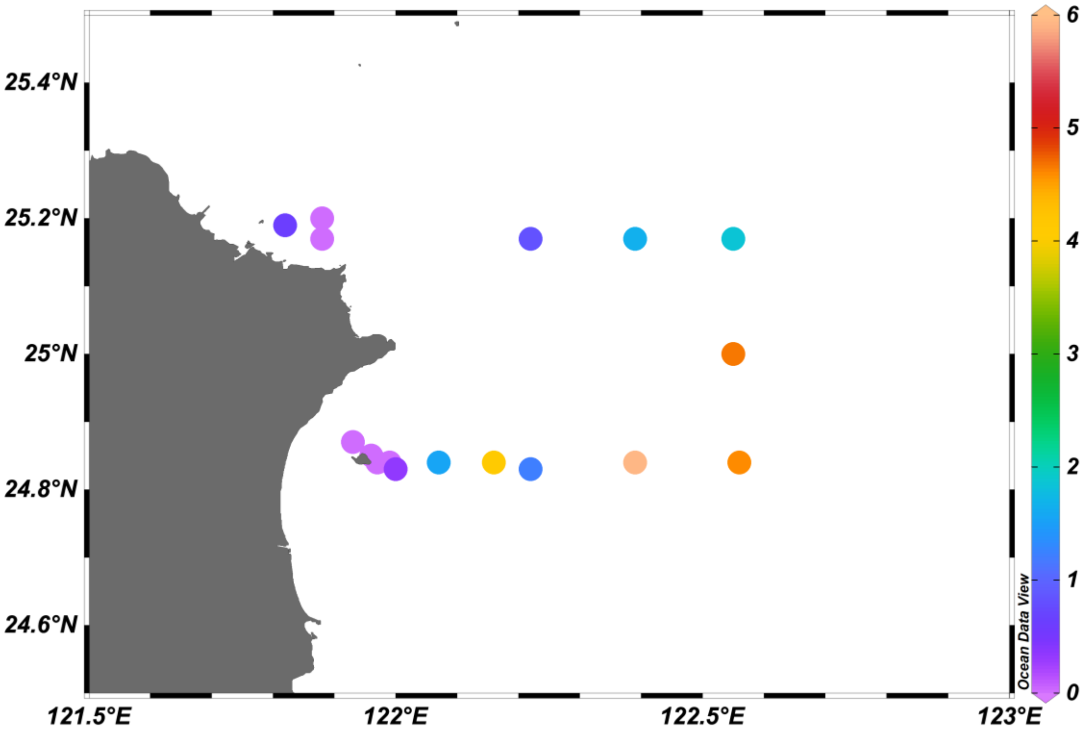

Figure S13. Distribution of *Copilia mirabilis* in the research area during our research.

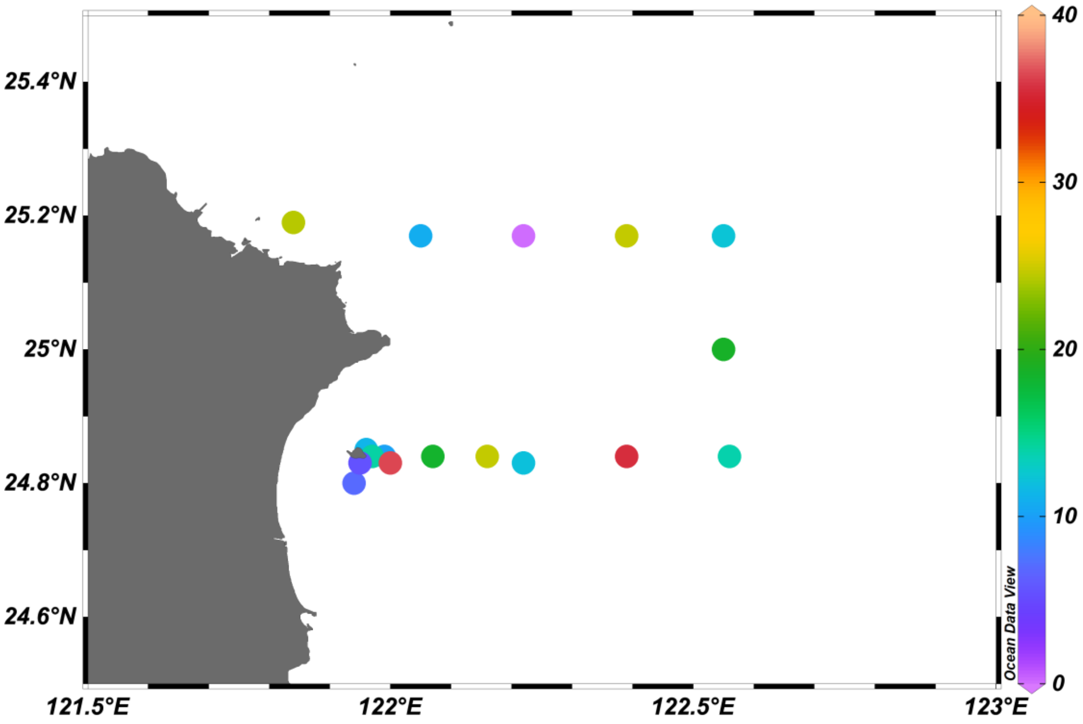

Figure S14. Distribution of *Corycaeus agilis* in the research area during our research.

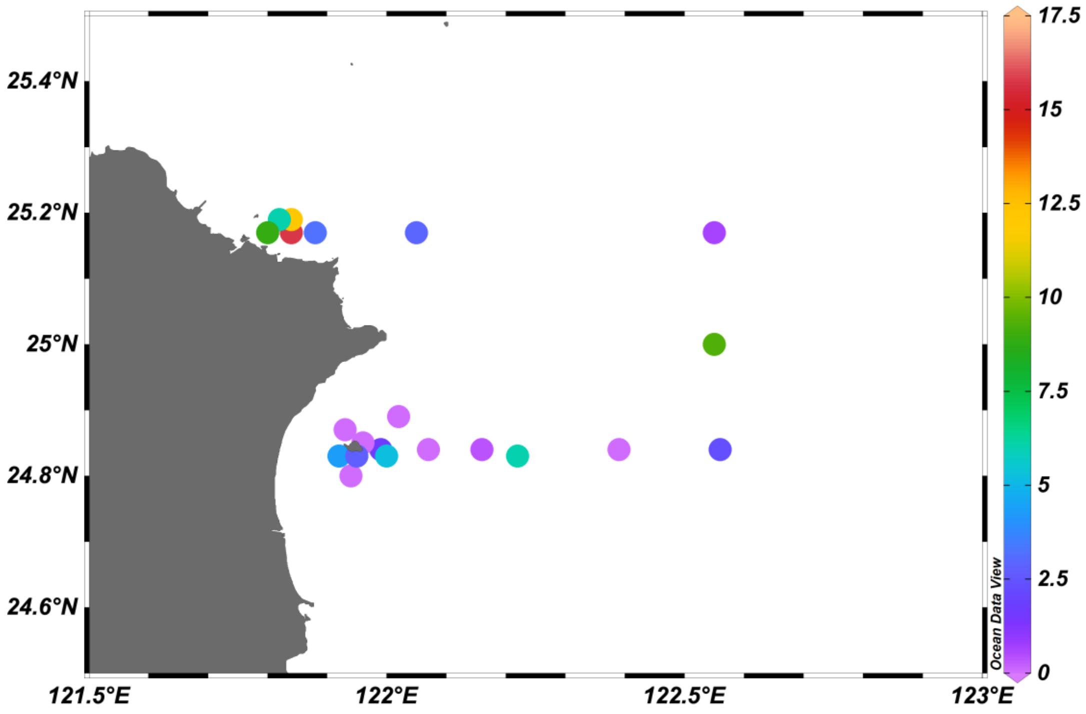

Figure S15. Distribution of *Corycaeus speciosus* in the research area during our research.

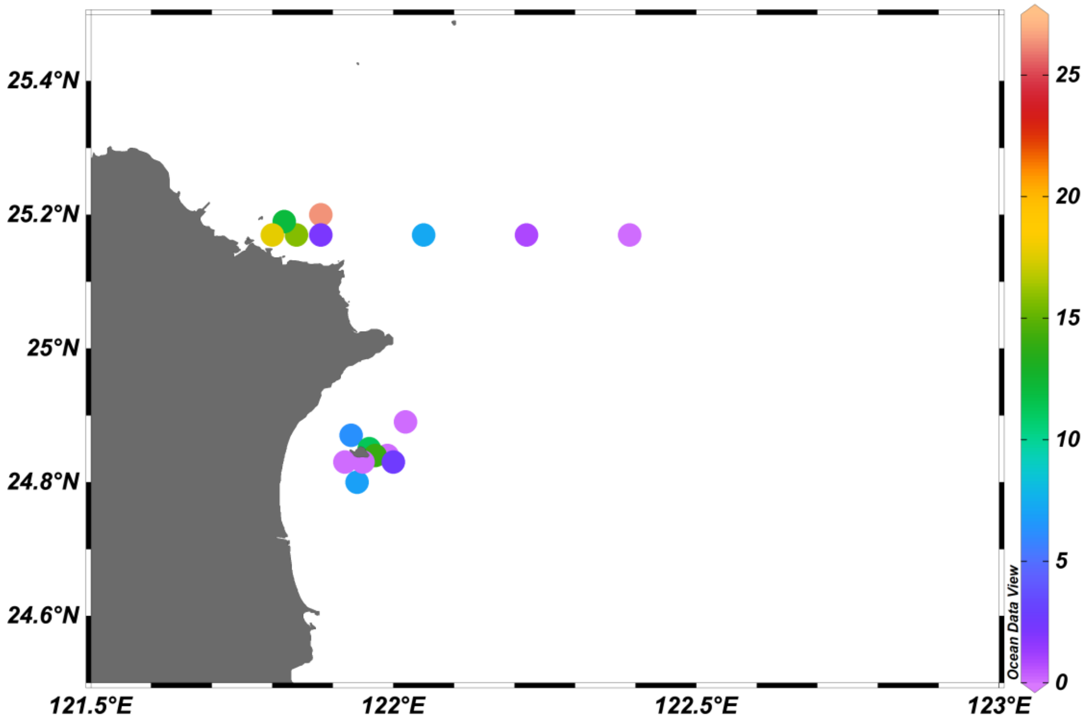

Figure S16. Distribution of the *Euchaeta concinna* in the research area during our research.

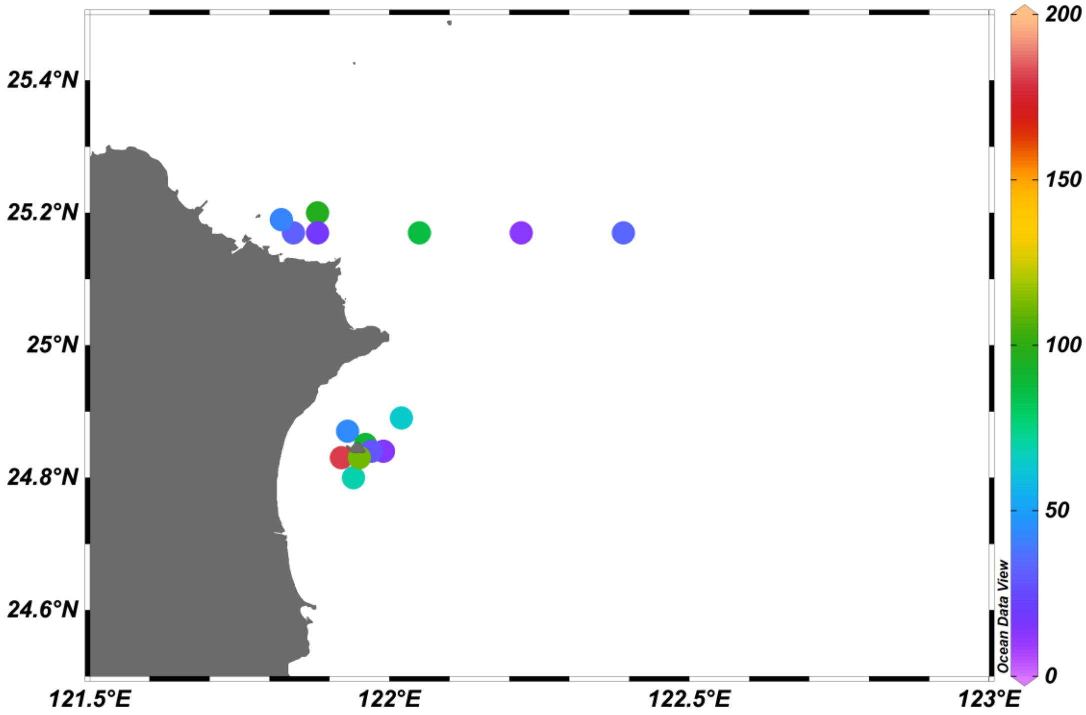

Figure S17. Distribution of the *Euchaeta* copepodites in the research area during our research.

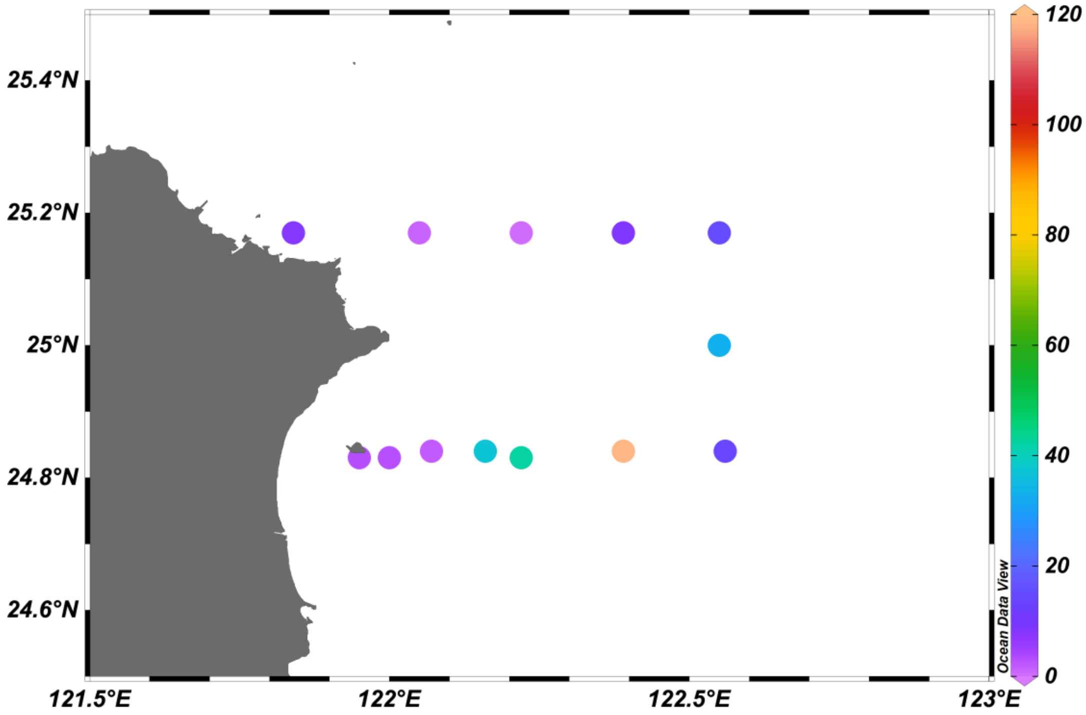

Figure S18. Distribution of *Farranula concinna* in the research area during our research.

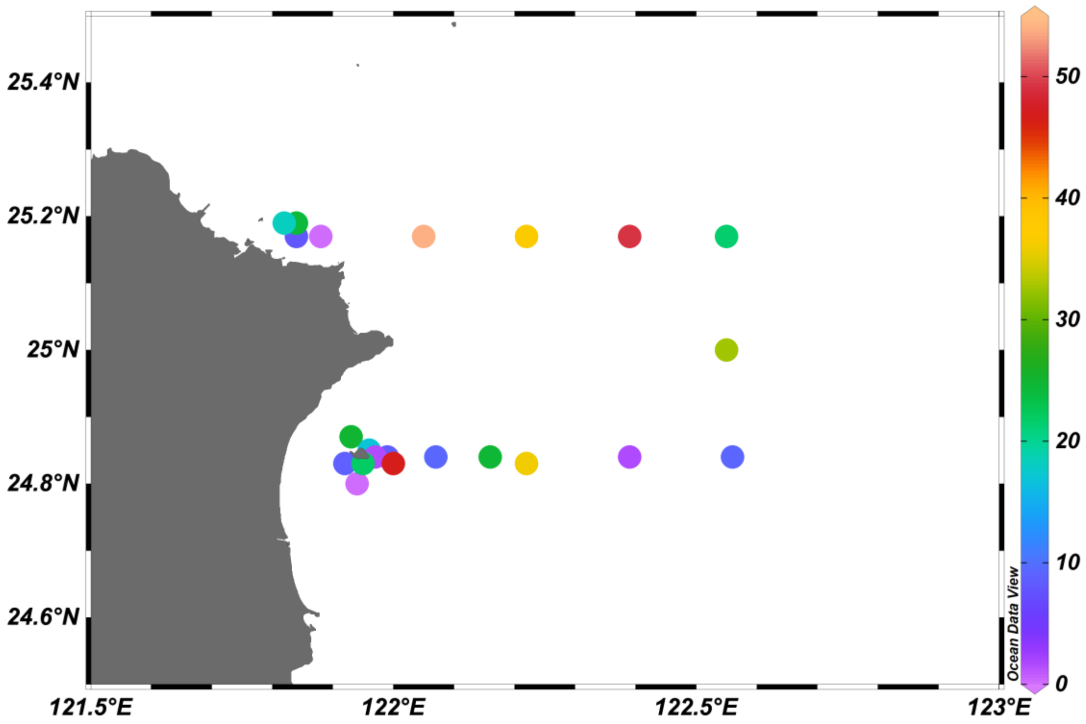

Figure S19. Distribution of *Farranula gibbula* in the research area during our research.

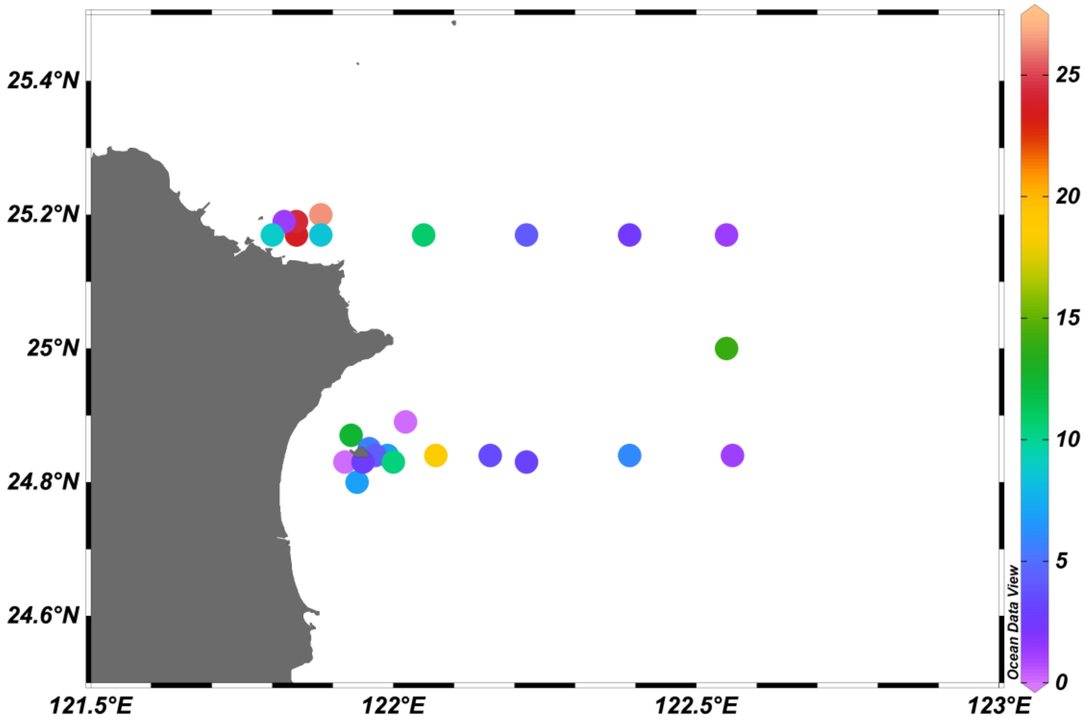

Figure S20. Distribution of *Macrosetella gracilis* in the research area during our research.

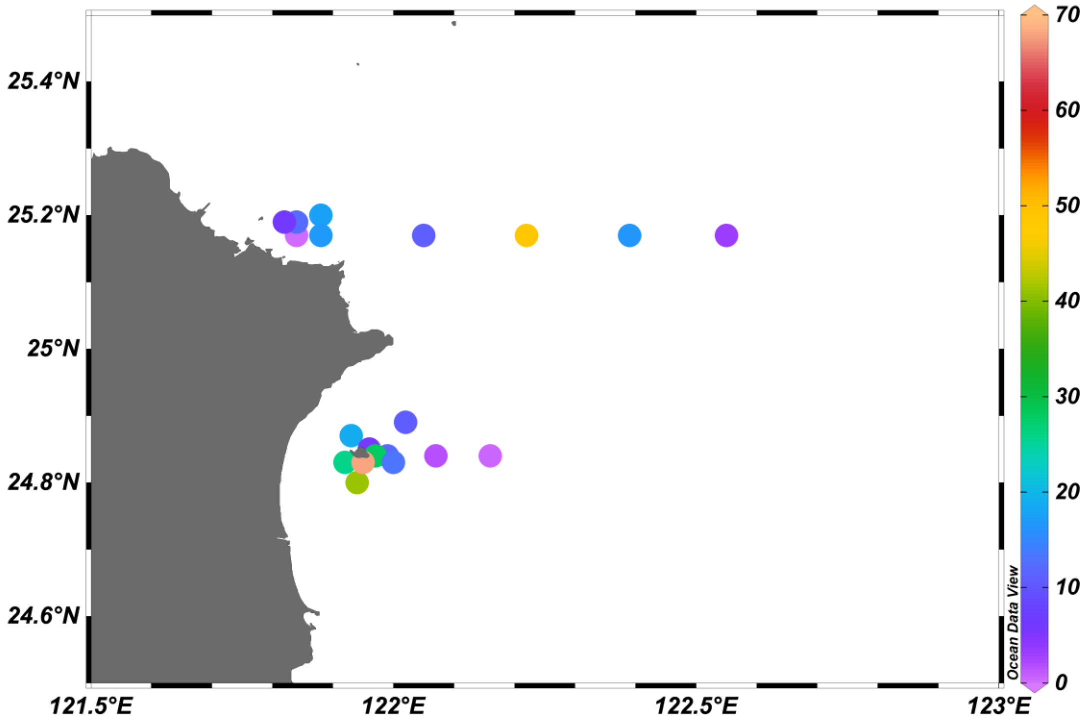

Figure S21. Distribution of *Oncaea clevei* in the research area during our research.

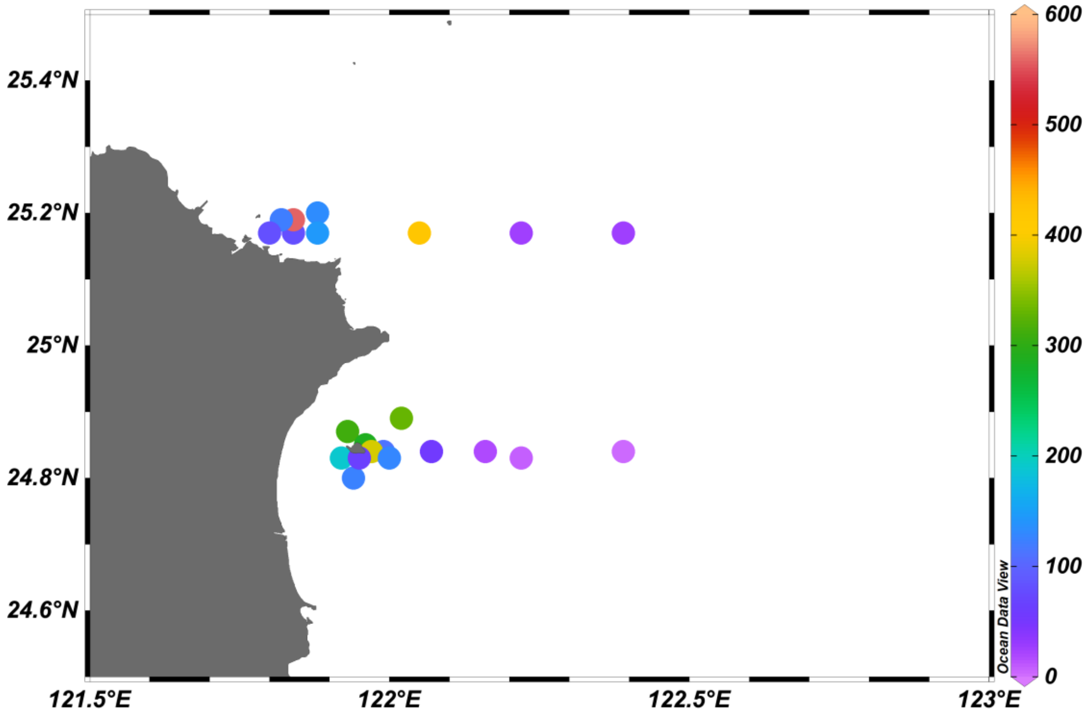

Figure S22. Distribution of *Paracalanus aculeatus* in the research area during our research.

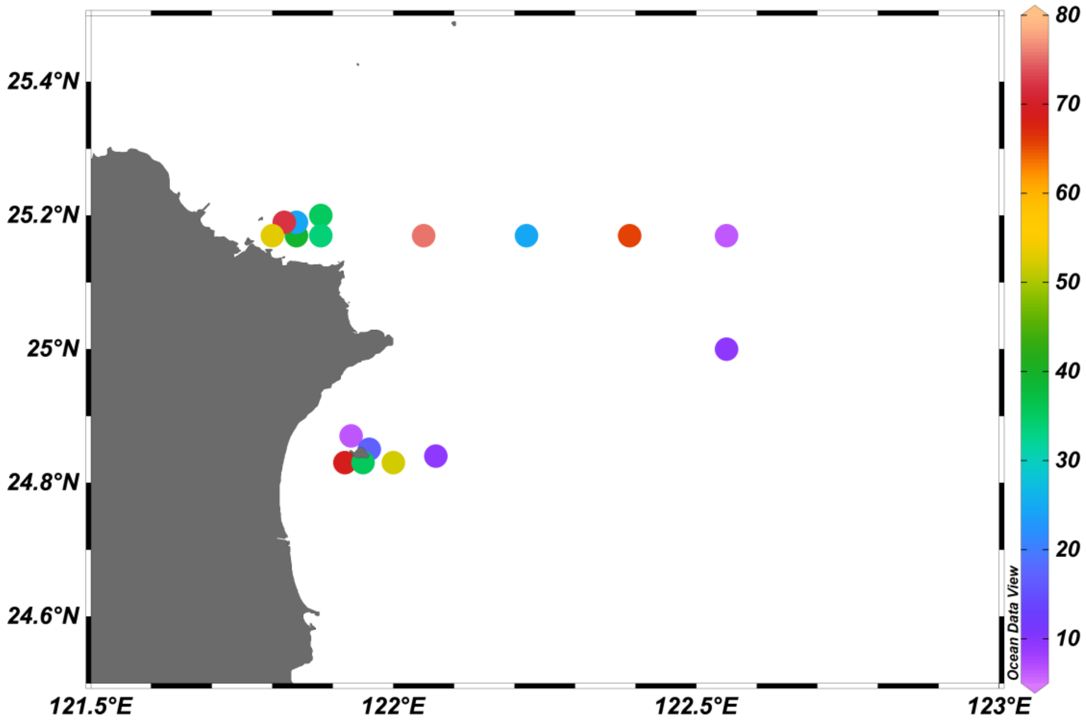

Figure S23. Distribution of *Paracalanus parvus* in the research area during our research.

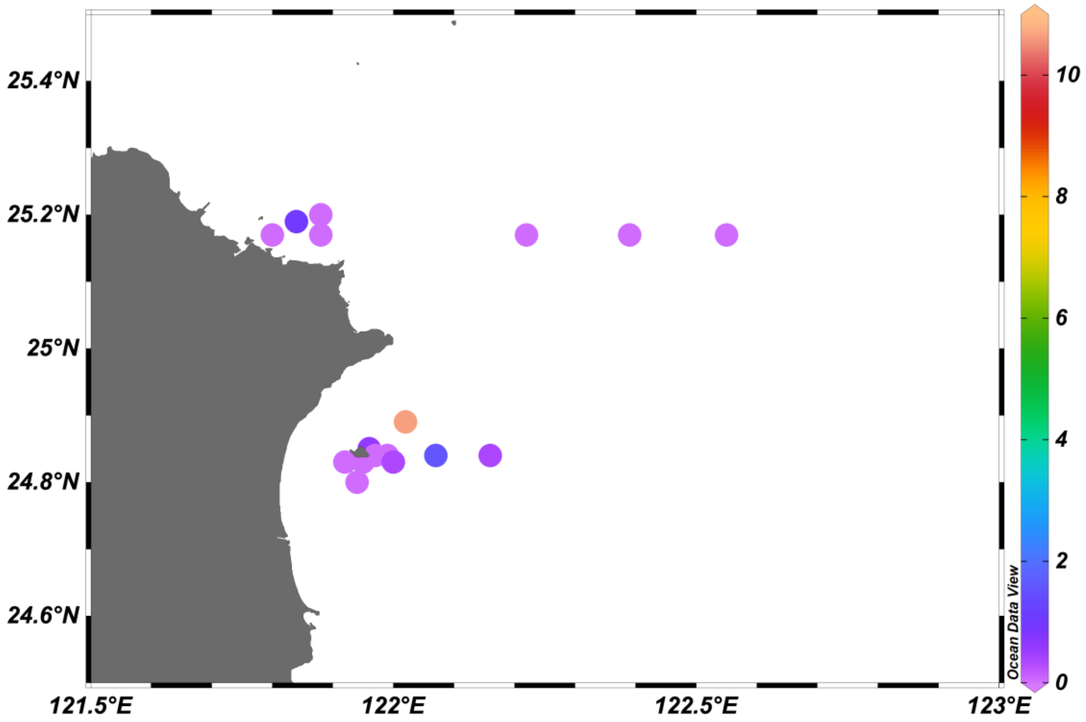

Figure S24. Distribution of *Paracandacia truncata* in the research area during our research.

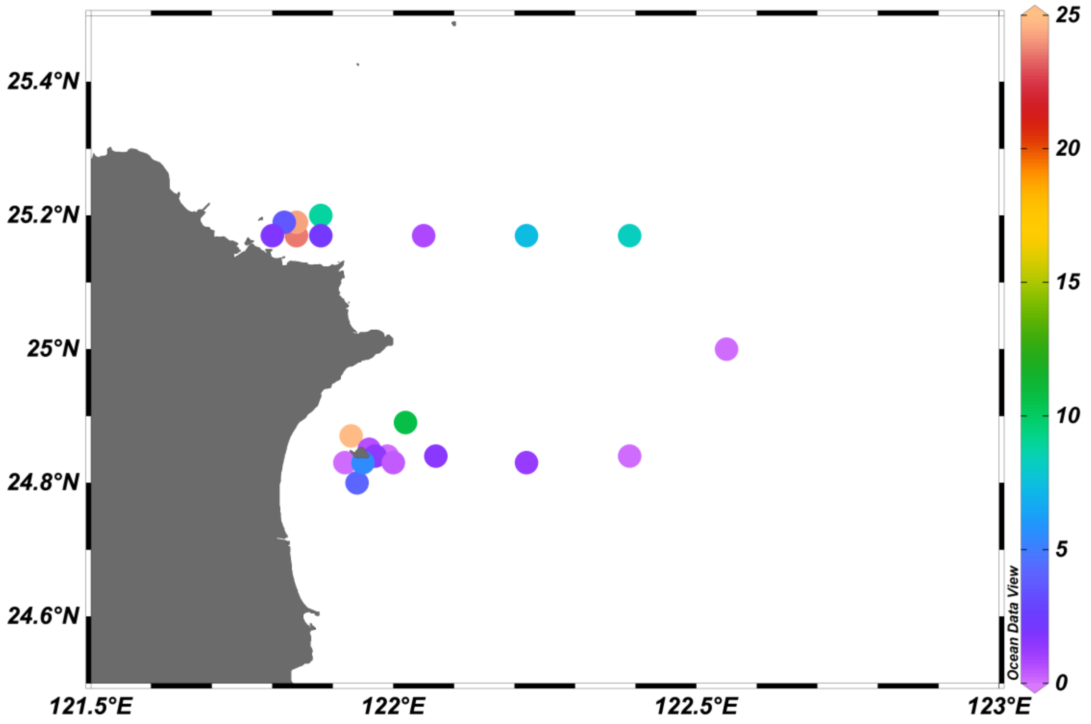

Figure S25. Distribution of *Temora discaudata* in the research area during our research.

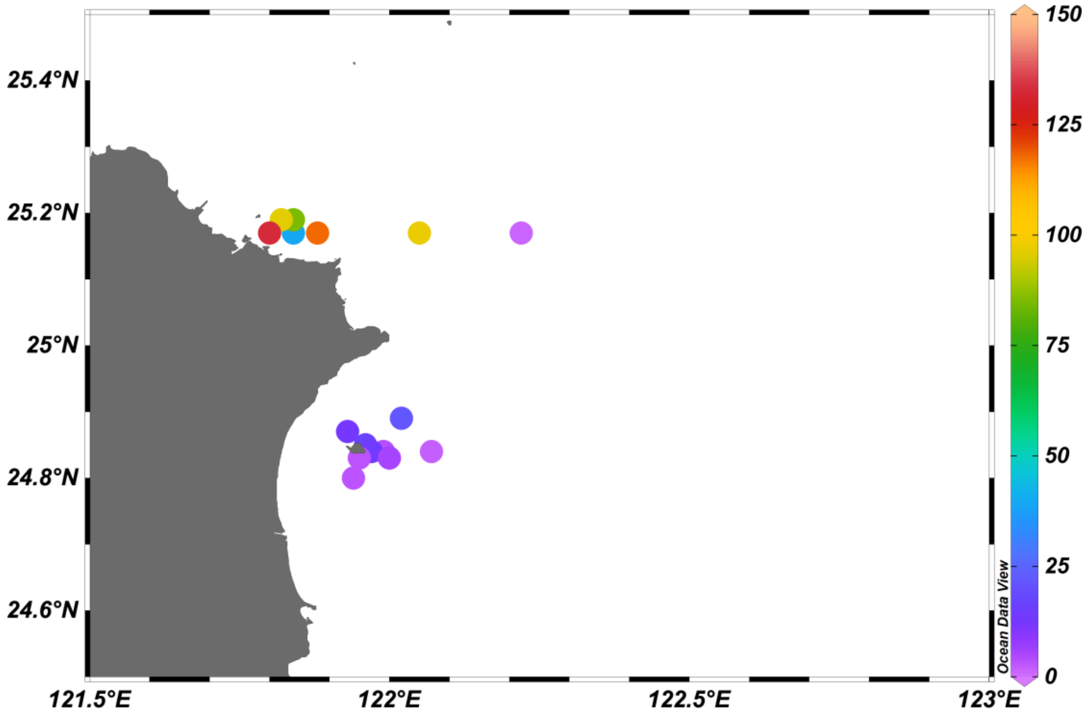

Figure S26. Distribution of *Temora turbinata* in the research area during our research.
